# A new self-adjustable glaucoma valve

**DOI:** 10.3389/fbioe.2024.1383459

**Published:** 2024-05-02

**Authors:** Soroush Rafiei, Julien Maxime Gerber, Stéphane Bigler, Nikolaos Stergiopulos

**Affiliations:** Laboratory of Hemodynamics and Cardiovascular Technology (LHTC), Swiss Federal Institute of Technology (EPFL), Lausanne, Switzerland

**Keywords:** glaucoma, self-adjustable valve, SAGDD, pressure-regulator, fluid-solid interaction, IOP management

## Abstract

**Introduction:** Glaucoma, the leading cause of irreversible blindness globally, affects more than 70 million people across the world. When initial treatments prove ineffective, especially for cases with high intraocular pressure (IOP), the preferred approach involves employing glaucoma drainage devices (GDDs).

**Methods:** This study introduces a novel self-adjustable glaucoma drainage device (SAGDD) designed to maintain IOP within the desired biological range (10 mmHg < IOP <18 mmHg) by dynamically modulating its fluidic resistance. Inspired by the starling resistor, we designed a circular valve with a thin, flexible membrane placed over the valve’s inlet and outlet. To achieve the ideal design for the SAGDD and optimize its parameters, we utilized fluid-solid interaction (FSI) numerical models and conducted parametric studies, wherein simulations demonstrated the validity of the concept. Subsequently, to confirm and validate the numerical results, we fabricated a SAGDD at a 3:1 scale and subjected it to *in vitro* testing.

**Results:** Our findings demonstrate that, on a 3:1 scale, a circular SAGDD with a diameter of 8.1 mm and a stainless-steel membrane with a thickness of 10 µm effectively maintained IOP within the target range when the membrane exposed to external pressures of 7.5 or 10 mmHg.

**Discussion:** In summary, our study establishes a strong foundation for further exploration of the potential efficacy of SAGDD as a promising treatment for glaucoma. The cost-effectiveness and simplicity of its design, devoid of costly instrumentation, hold considerable promise in addressing the challenges associated with glaucoma.

## 1 Introduction

Over 70 million individuals worldwide suffer from glaucoma, the main cause of permanent blindness worldwide (Lee et al., 2020). According to the predictions, the estimated number of people with glaucoma will rise from around 76 million in 2020 to more than 110 million in 2040 as a result of population aging and increasing lifespan (Tham et al., 2014; Wang et al., 2019). One of the main risk factors for glaucoma is a rise in intraocular pressure (IOP >21 mmHg), which causes progressive optic nerve damage and visual field loss.

The only established method of halting the development of visual loss in glaucoma is to reduce IOP, which is the major focus of the current treatment approaches. Drugs and laser therapy are usually the primary treatments (Conlon et al., 2017; Yadav et al., 2019) and surgery is necessary when the primary interventions are insufficient to decrease IOP within the target pressure levels (Lusthaus and Goldberg, 2019). Implantation of glaucoma drainage devices (GDDs), trabeculectomy, deep sclerotomy, and minimally invasive glaucoma (MIGS) procedures are a few examples of filtering surgeries used to treat glaucoma. Each of the available surgical techniques is based on the same fundamental idea: bypassing/enhancing the eye’s normal outflow routes to provide a different path for aqueous humour to successfully drain out of the anterior chamber and thus decrease IOP (Gedde et al., 2011; Conlon et al., 2017). Given that they have demonstrated lower complication rates when compared to conventional trabeculectomy, GDDs and MIGS have been used more frequently in the past 20 years (Gedde et al., 2005; Gedde et al., 2009; Desai et al., 2011; Gedde et al., 2012; Vinod et al., 2017); however, MIGS, such as the Preserflo MicroShunt, along with similar implants like Xen, are primarily employed for patients with mild-to-moderate glaucoma (Gurnani and Tripathy, 2023). Despite their effectiveness, these implants are associated with certain complications, including device obstruction, hypotony, hyphema, and the need for secondary surgical interventions (Vinod and Gedde, 2021; Gurnani and Tripathy, 2023; Traverso et al., 2023; Home Page). GDDs present a viable alternative for managing complex glaucoma characterized by a heightened risk of failure in conventional filtering surgeries. These complex cases encompass a spectrum of conditions including aphakia or pseudophakia, neovascular glaucoma, trauma-related glaucoma, uveitis-related glaucoma, epithelial downgrowth, iridocorneal endothelial syndrome, vitreoretinal disorders, and cases post-penetrating keratoplasty (Assaad et al., 1999). In such challenging scenarios, where conventional methods may not suffice, GDDs emerge as a preferred option, offering distinct advantages over alternative approaches, particularly for advanced-stage glaucoma (Gurnani and Tripathy, 2023).

The tube and plate concept developed by Molteno has been largely maintained in the design of modern GDDs (Molteno, 1969). In these devices, the aqueous humour is drained from the anterior chamber via a tube and a plate placed in the subconjunctival area on the equatorial part of the eye. Following the Molteno, successive devices made minor changes to the GDD design in an attempt to increase surgical success and prevent failures. Georges Baerveldt invented the Baerveldt GDD in 1990 (Poelman et al., 2020). It was made out of a silicone tube linked to a malleable barium impregnated silicone plate that came in a variety of sizes. The main problem, however, with valve-less Molteno and Baerveldt GDDs was hypotony, indicated by excessively low IOP in the early post-operative phase (Dixon et al., 2020). To address hypotony, Marteen Ahmed launched the Ahmed glaucoma valve in 1993 (Coleman et al., 1995). Based on the venturi effect, the Ahmed valve has two silicone membranes that are pre-tensioned to open at an IOP of 8 mmHg. The Ahmed valve became the most used GDD in the world, however, its long-term performance is not optimal because its valve structure adds constant fluidic resistance to flow leading to high IOPs (>21 mmHg) and failures (Christakis et al., 2016). To address the problems in the previous GDDs, the eyeWatch implant (Rheon Medical SA, Lausanne, Switzerland), the world’s first commercially available adjustable glaucoma implant, was developed at the laboratory of hemodynamics and cardiovascular technology (LHTC). The eyeWatch implant includes an eccentrically rotatable magnetic disk which is used to apply variable compression on an internal deformable tube, thereby altering its fluidic resistance to keep the IOP within the clinically desired range (Villamarin et al., 2014a; Villamarin et al., 2014b). The readout of the magnetic disk rotational position and the rotation of the disk is achieved with the eyeWatch Pen, an external hand-held device that is used as a control unit. Despite the desirable efficacy demonstrated in clinical trials (Roy and Mermoud, 2024), including total avoidance of hypotony-related issues, the eyeWatch has two major shortcomings: first, it is not a self-adjustable valve, needing human intervention to adjust the valve resistance and, second, because of its complicated mechanism, it is very costly to manufacture, making the end-user price is quite expensive in comparison to all other GDDs in the market.

In this study, a novel self-adjustable glaucoma drainage device (SAGDD) that addresses the previously mentioned issues—namely, lack of self-adjustability and high manufacturing costs, is introduced. This new implant keeps IOP within the target range by self-adjusting its resistance. This new SAGDD is designed to provide significant resistance to prevent the IOP from falling below a specified value (IOP <10 mmHg) in the early postoperative period and, as time advances and the bleb forms around the drainage plate increasing outflow resistance, the SAGDD resistance decreases sufficiently in order to keep IOP levels within the target range while, at the same time, preventing long-term hypertony (in this paper, hypertony refers to intraocular pressures exceeding 18 mmHg).

## 2 Materials and methods

### 2.1 Numerical simulations and parametric studies

The assessment and optimization of valve functionality were initially performed through numerical simulations and subsequently validated experimentally. The analysis of valve flow necessitated the incorporation of fluid-solid interaction (FSI) capabilities, primarily due to the pivotal role played by the flexible membrane deformation in the function of the valve. A similar numerical approach has been employed in various research papers (Fanelli et al., 2019; Paz et al., 2021). This comprehensive study involved conducting a two-way FSI analysis, aimed at gaining insights into the dynamics of fluid flow within the microvalve, the deformations exhibited by the membrane (solid domain) in response to fluid flow (fluid domain) forces, and the resulting changes in microvalve performance depending on variations in design parameters. The FSI simulations were conducted utilizing the commercial ANSYS software package (ANSYS, Canonsburg, United States).

In the solid domain of this study, Ansys Mechanical was utilized to conduct the analysis, enabling large deformation to be incorporated for the purpose of obtaining more accurate and realistic results, albeit with an increase in simulation time. Furthermore, simulations were conducted under steady-state conditions using the implicit solver, which is well-suited for seeking steady-state solutions. A quadratic nonlinear mechanical mesh was employed for membranes. An appropriate time step, ranging from 0.001 to 0.1 s, was automatically selected by the software, with a step end time set at 1s.

In the fluid domain, water, characterized as an incompressible and viscous fluid, was selected as the working medium. As boundary, a constant physiological flow rate of 2 μL/min at the inlet was applied, which reflects the average production rate of aqueous humor in the human (Sunderland and Sapra, 2023), and varying pressures for the outlet to simulate the increased resistance to aqueous humour drainage associated to distal fibrosis formation. Because of the very small flow rates and dimensions, Reynolds number was very low and flow was modeled as laminar in the entire flow field. Owing to the inherent symmetry of the design and to optimize computational efficiency, only half of the complete valve geometry was utilized in the simulations. The simulation also employed a dynamic mesh approach within Fluent software to accommodate changes in the fluidic domain as a response to membrane deformation. Control parameters such as the number of iterations and relaxation factors were manually adjusted to ensure convergence in each case. Finally, for the coupling system, the minimum and maximum iterations were set to 10 and 100, respectively.

Understanding the impact of design parameters on valve performance is paramount for the development of a functional valve. Our investigations consist of an examination of membrane dimensions (thickness and diameter) and the applied external pressure on the membrane. Given the constraints imposed by surgical considerations, a preference for smaller valve dimensions is evident. In this context, we specifically focused on assessing the influence of membrane diameter on its maximum deformation, aiming to identify an optimal diameter for subsequent analysis. Accordingly, we selected membranes with diameters of 2, 2.7, and 3.4 mm for in-depth examination. Stainless steel was chosen as the material for the membrane due to its established use as a biocompatible material in implantable ophthalmic devices (Hermawan et al., 2011; Zhang et al., 2015). The membrane thickness options of 3, 5, and 10 µm were specifically chosen as they are pre-determined values provided by a reliable supplier. This deliberate choice allowed for a comprehensive exploration of the parameter space within the study. In addition to the specified design parameters, considering that aqueous humor production rates may vary among individuals, a sensitivity analysis for this parameter was conducted numerically to ensure the functionality of the device under various circumstances. To achieve this, the valve’s behavior was examined by setting the flow rate to 1, 2, and 3 μL/min. This corresponds to flow rates of 27, 54, and 81 μL/min in the 3:1 scale-up prototype. This analysis provides insights into how the device responds to different aqueous humor production rates, ensuring its effectiveness across a range of conditions. The list of the parameters utilized in this study is summarized in Table 1.

**TABLE 1:** Parameters utilized in the numerical simulations.

Parameter	Values
Membrane/valve dimeter	2, 2.7, 3.4 (mm)
membrane thickness	3, 5, 10 (µm)
external pressure	0, 7.5, 10 (mmHg)
flow rate	1, 2, 3 (µL/min)

### 2.2 Experimental setup

**FIGURE 1:**
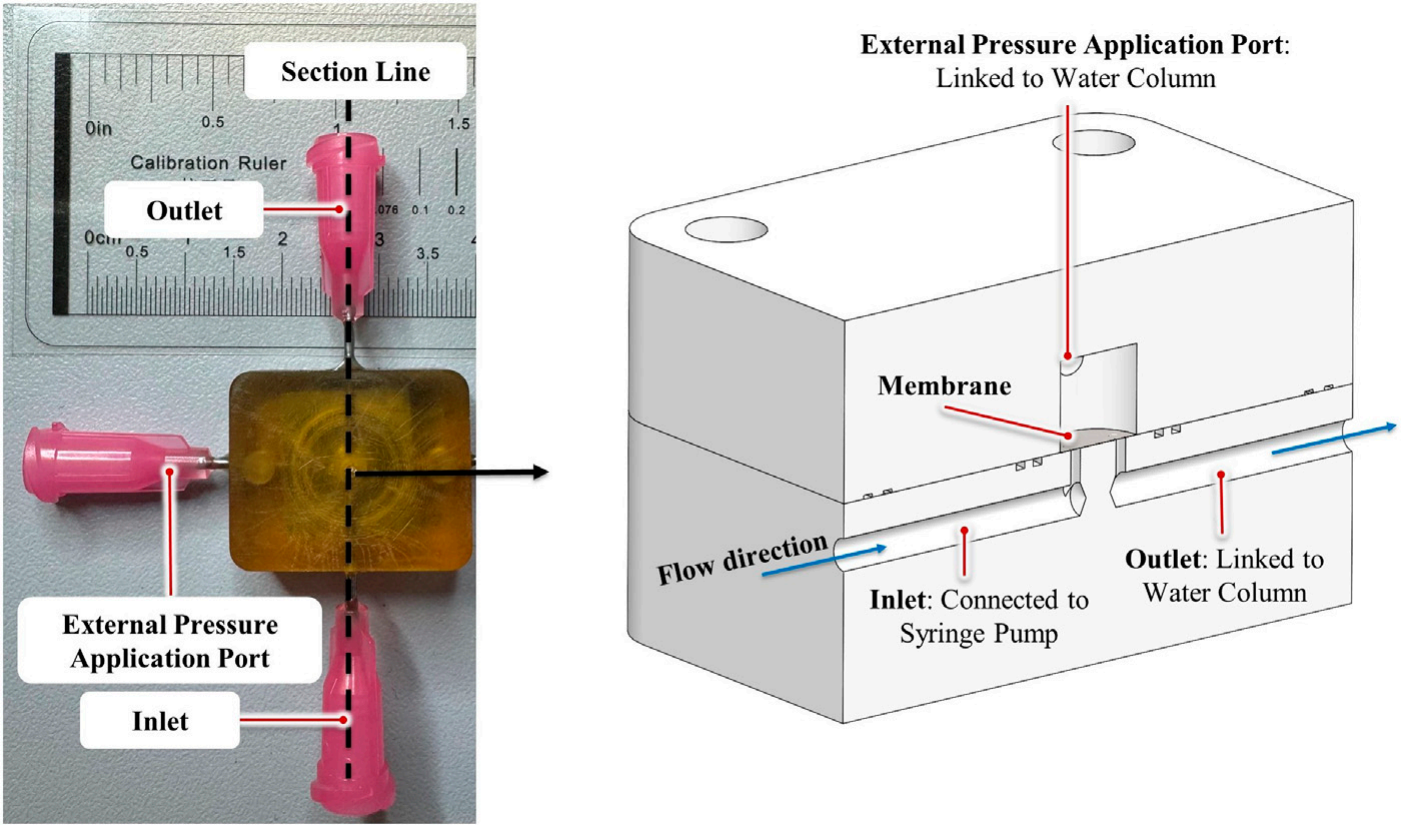
The SAGDD and its cross-section view (right).

Based on the simulations and the design optimization work, a valve with the desired functionality was developed. To translate the virtual design into a physical reality, a 5- axis micro-computer numerical control (CNC) machine (DATRON AG, Mühltal, Germany) was employed to manufacture the valves. To do this, an appropriate input for the CNC device was generated using Mastercam 2021 (CNC Software, Inc., Tolland, United States) software from Solidworks 2020 (Dassault Systèmes SolidWorks Corporation, Waltham, United States) models. To enhance manufacturability, ease of handling, and reduce sensitivity to tool precision, a scaled-up 3:1 prototype of the valve was fabricated (Figure 1).

When scaling the system, it is essential to estimate the corresponding flow rate, which was achieved through dimensional analysis in this study. Considering that the pressure-drop ( ∆P ) in the valve is a function of geometrical parameters ( $\left.l_{1},l_{2},l_{3},\ldots ,l_{n}\right)$ , material properties of the membrane ( $\left.E\right)$ , fluid viscosity ( μ ) and flow rate ( $\left.Q\right)$ , this relationship can be expressed as Eq. 1:

$$∆P=f\left(l_{1},l_{2},l_{3},\cdots ,l_{n},h,d,E,\mu ,Q\right)$$ (1)

Then, according to the Buckingham π theorem (Buckingham, 1914), Eq. 2 can be expressed in terms of the nondimensional terms:

$$\frac{P}{E}=\emptyset \left(\frac{l_{1}}{d},\frac{l_{2}}{d},\frac{l_{3}}{d},\cdots ,\frac{l_{n}}{d},\frac{h}{D},\frac{Q.\mu }{E.d^{3}}\right)$$ (2)

Given that the Young’s modulus (*E*) remains the same between the model and prototype, it follows that the pressure drop will also be the same, provided that the design conditions between model and prototype are satisfied. In accordance with the design conditions, the flow rate in the prototype can be expressed by Eq. 3:

$$Q_{m}={Q.\left(\frac{d_{m}}{d}\right)}^{3}=Q.{\left(\frac{3}{1}\right)}^{3}=27.Q$$ (3)

The standard flow rate in the actual-size system is 2 μL/min, a typical production rate of aqueous humor in the eye. Therefore, in the 3:1 scale model, the flow rate is 27 times greater, amounting to 54 μL/min.

**FIGURE 2:**
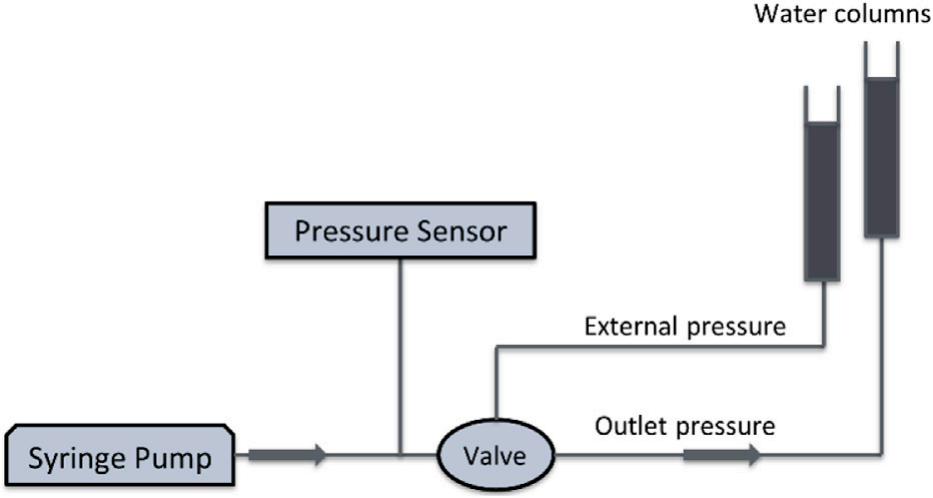
The schematic of the measurement setup of the SAGDD.

The valve’s pressure response was tested using the experimental setup seen in Figure 2. A syringe pump delivered water through the microvalve at a rate of 54 μL/min. Upstream pressure was read by pressure sensor. The outlet of the valve was connected to a water column, the height of which can be adjusted to generate different levels of distal pressure and imitate the increased resistance to aqueous humor drainage associated with fibrosis formation. To apply external pressure on the membrane, a second water column was used. Proximal (inlet) pressure was measured at various outlet pressures, ranging from 0 to 35 mmHg, and various external pressures ranging from 0 to 10 mmHg. The materials and equipment employed for both manufacturing and testing the valves are listed in Table 2.

**TABLE 2:** The materials and equipment used to manufacture and test the valves.

Materials/Equipment	Description
5-axis CNC machine	DATRON C5
Syringe pump	WPI AL-1000
Pressure sensor	Cynergy3 IPSL-G0050-5, Pressure Transducer 0–50 mbar
Valve material	PMMA
Membrane	Stainless steel 1.4404 foil, thickness = 10 μm

## 3 Results

### 3.1 Self-adjustable glaucoma drainage device

**FIGURE 3:**
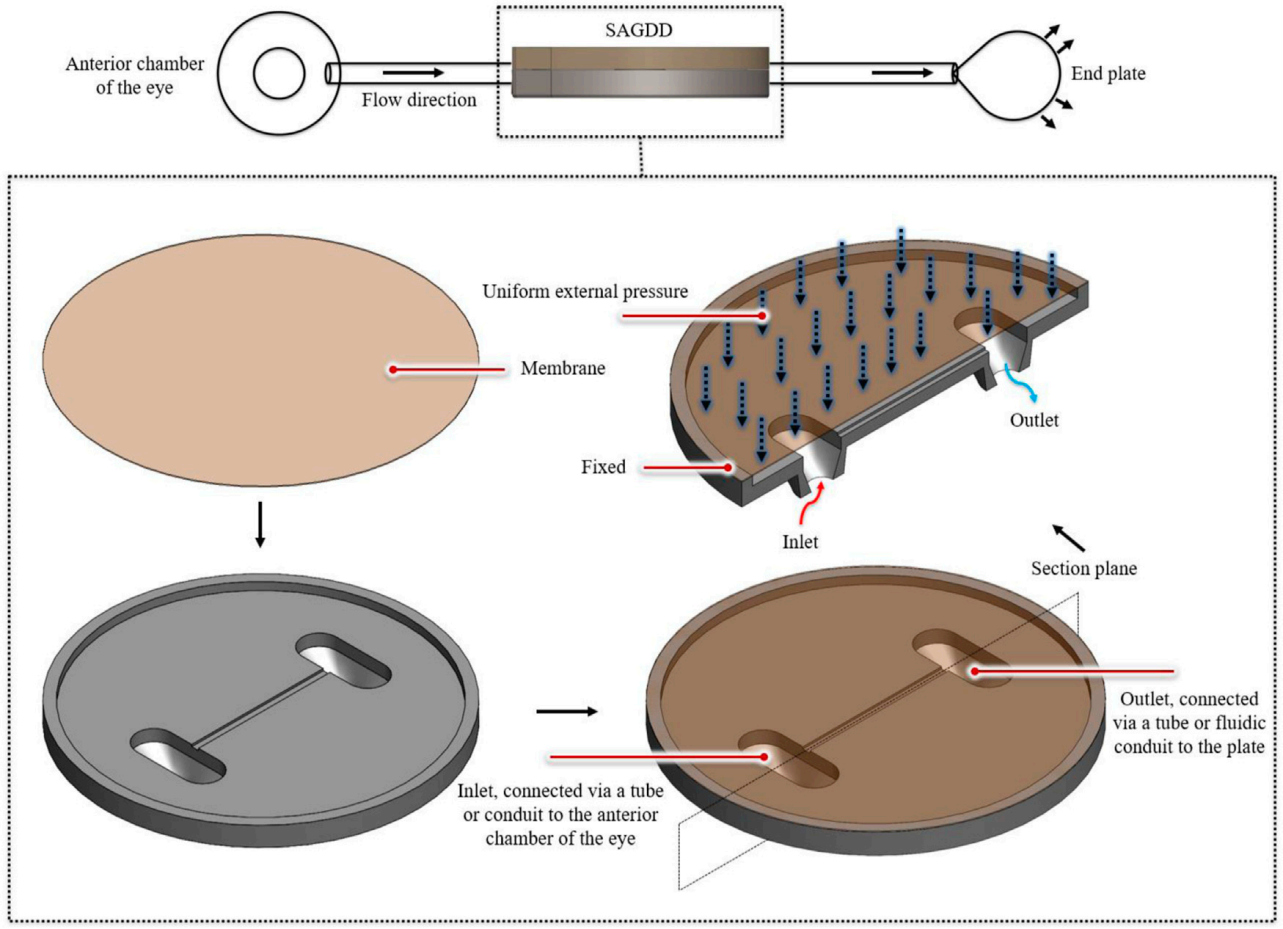
Schematic representation of the innovative Self-Adjustable Glaucoma Drainage Valve. In the top-left sectional view, the key components of the novel SAGDD are depicted, illustrating the inlet connected via a tube or conduit to the anterior chamber, the outlet connected via a tube or fluidic conduit to the plate, and the external pressure regulation mechanism.

The design of the SAGDD, intended to act as a pressure regulator, is depicted in Figure 3. Inspired by the functioning principles of a Starling resistor concept, the circular valve features a very thin metallic flexible membrane, that covers the inlet and outlet of the valve, creating a certain resistance to flow. An external pressure, as depicted in the figure, is applied to the membrane to ensure the closure of the valve when IOP is below a certain threshold. This helps avoid hypotony in the early postoperative period when the bleb is not formed yet and distal pressure is close to zero. When either inlet (upstream or intraocular) pressure or outlet (distal) pressure increases, the valve bulges out reducing resistance to flow and, in turn, reducing pressure. This effect ensures that the inlet pressure (IOP) remains relatively stable. In the early post-operative period, when there is no fibrosis and distal pressure is practically zero, the membrane is less deformed and the fluidic resistance is high, keeping the upstream pressure high and avoiding hypertony. This innovative design, was previously patented by members of the LHTC (Stergiopulos, 2009).

### 3.2 Numerical simulations and parametric studies

**FIGURE 4:**
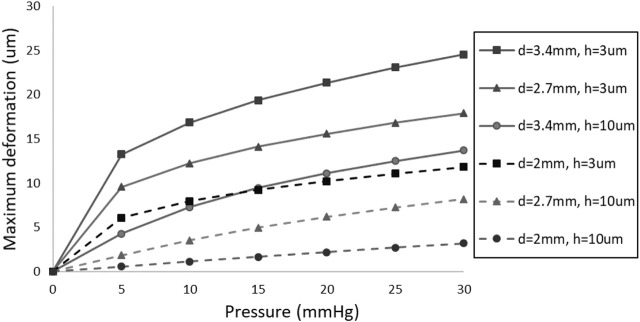
The effect of membrane thickness (h) and diameter (d) on its maximum deformation.

Understanding the impact of membrane diameter and thickness on their maximum deformation, as illustrated in Figure 4, is crucial, since the deformability of the membrane significantly influences valve resistance. The deformation of the membrane occurs within the central region of the membrane. This is because the edges, being fixed, constrain the movement, causing the central portion of the membrane to undergo the most significant deformation in response to the applied pressure. The results indicate a substantial increase in maximum membrane deformation when the membrane diameter increases from two to 3.4 mm. Furthermore, increasing the thickness from 3 to 10 µm results in a significant reduction in maximum deformation. The key observation from this figure is that the deformation *versus* pressure curve is non-linear, demonstrating relatively high compliance at low pressures (0–15 mmHg), which falls within our primary pressure range of interest. Following a comprehensive examination of these factors and considering constraints related to manufacturing and surgery, a diameter of 2.7 mm was selected for subsequent FSI simulations. By altering the thickness at this diameter, a broad spectrum of membrane deformability can be achieved, ranging from an easily deformable membrane to a hard, less deformable one.

**FIGURE 5:**
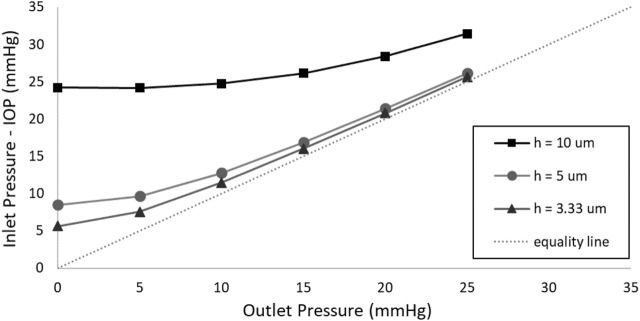
The effect of membrane thickness on the SAGDD behavior.

Figure 5 illustrates the impact of membrane thickness on valve functionality, considering outlet pressures ranging from 0 to 25 mmHg. It is important to mention that, owing to the unavailability of a 9 µm thickness membrane—a crucial component for accurately scaling up the valve with the original 3 µm membrane by a factor of 3—a membrane with a thickness of 3.33 µm was utilized in this simulation since a 10 µm membrane was available. The simulated valve retains the same geometric configuration as depicted in Figure 3, and it is essential to emphasize that no external pressure was applied to the membrane during this analysis.

It is evident in Figure 5 that, irrespective of the membrane thickness, the pressure-drop/resistance in the valve decreases as the outlet pressure of the valve increases. For outlet pressures exceeding 15 mmHg, the pressure-drop in the valve with a 3.33 thick membrane is negligible (less than 1 mmHg), unlike the valve with a 5 and 10 µm thick membrane. In the range of outlet pressures from 0 to 15 mmHg, reducing the membrane thickness from 10 to 3.33 µm notably lowers IOP, with the most significant difference observed at 0 outlet pressure, decreasing it from approximately 24.2 to 5.6 mmHg.

The valve with the 3.33 µm thickness membrane remains nearly fully open (very low pressure-drop) at distal pressures exceeding 15 mmHg, effectively minimizing the risk of hypertony in the late postoperative period, and it outperforms the 5 μm and 10 µm membranes in terms of hypertony prevention. However, it faces a challenge in offering adequate resistance at 0 mmHg distal pressure, leading to hypotony. To address this issue, an external pressure was applied to the membrane, successfully resolving the problem and achieving the ideal SAGDD.

### 3.3 The ideal SAGDD

**FIGURE 6:**
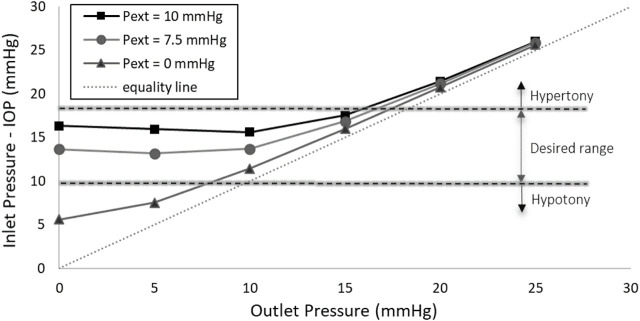
Inlet Pressure vs. Outlet Pressure at different external pressures (Pext) in the real-size numerical model. The application of 7.5 or 10 mmHg external pressure on the membrane effectively maintains the IOP within the desired range.

Figure 6 illustrates the valve with a 3.33 µm membrane thickness and a 2.7 mm diameter stainless steel membrane, exposed to external pressures of 7.5 and 10 mmHg, as well as without applying external pressure. As mentioned earlier, an ideal glaucoma implant should effectively mitigate the risk of hypotony by providing substantial fluidic resistance when distal pressure is low, the resistance being automatically adjusted as to keep IOP fairly constant when distal resistance increases due to bleb formation and fibrosis. It is evident that external pressures of 7.5 and 10 mmHg primarily impact the valve at lower distal pressures (increasing the IOP by 8 and 10.7 mmHg when the outlet pressure is set to 0, respectively). However, they do not add significant resistance to the valve when the outlet pressure exceeds 15 mmHg (increasing the IOP by less than 0.9 and 1.6 mmHg, respectively). This implies that while aiding in hypotony prevention, external pressure does not adversely affect the long-term hypertony prevention performance of the valve. According to this figure, external pressures of 7.5 and 10 mmHg successfully maintain the inlet pressure (IOP) within the desired range, with 7.5 mmHg external pressure performing slightly better in hypertony prevention.

### 3.4 Validation of numerical results

**FIGURE 7:**
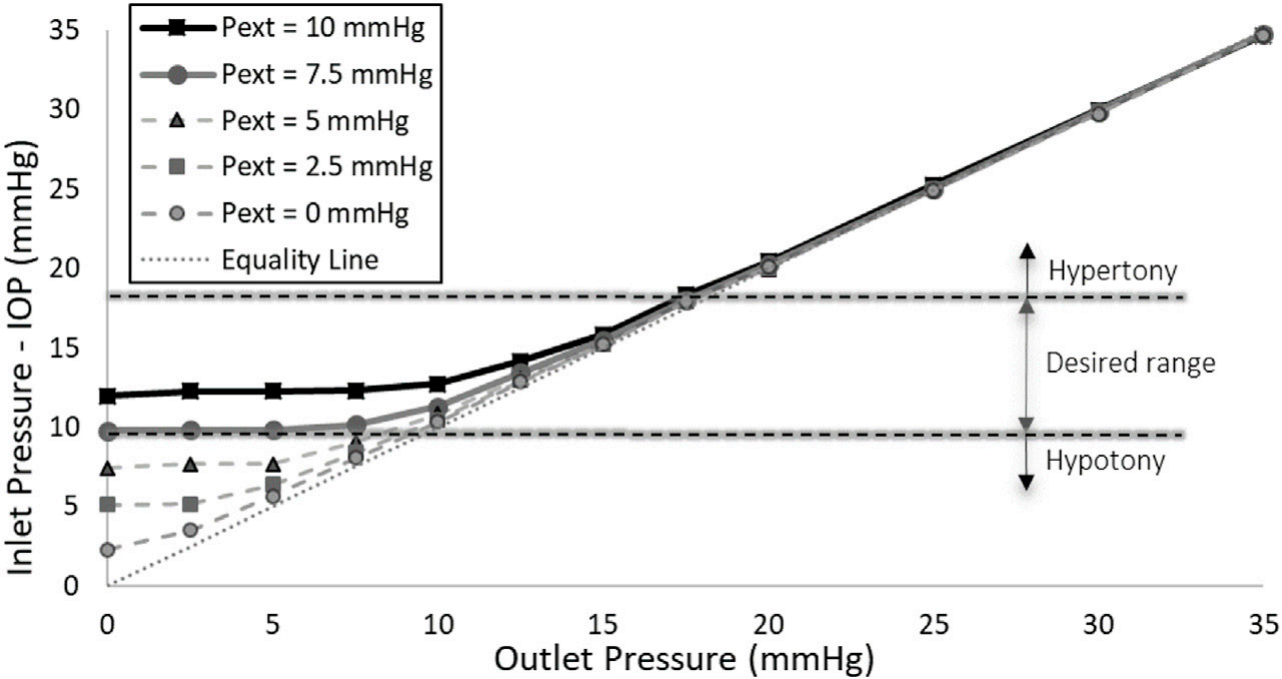
Inlet Pressure vs. Outlet Pressure at various external pressures (Pext) in the scaled-up prototype. The application of 7.5 or 10 mmHg external pressure on the membrane effectively maintains the IOP within the desired range.

The experimental findings for a valve with a 3:1 scale, featuring an 8.1 mm diameter and a 10 µm thick stainless-steel membrane, are presented in Figure 7. Similar to numerical simulation results, this figure shows that when the membrane is subjected to external pressures of 7.5 and 10 mmHg, the valve effectively maintains the IOP within the target range, demonstrating its suitability as an ideal SAGDD. At these external pressures, when the outlet pressure is 0, the inlet pressure of the valve is relatively high due to the resistance provided by the valve. However, as the outlet pressure increases, simulating an increase in fibrotic resistance, the valve’s resistance markedly decreases. Eventually, it approaches zero when outlet pressures exceed 15 mmHg.

### 3.5 Sensitivity to the inlet flow rate

**FIGURE 8:**
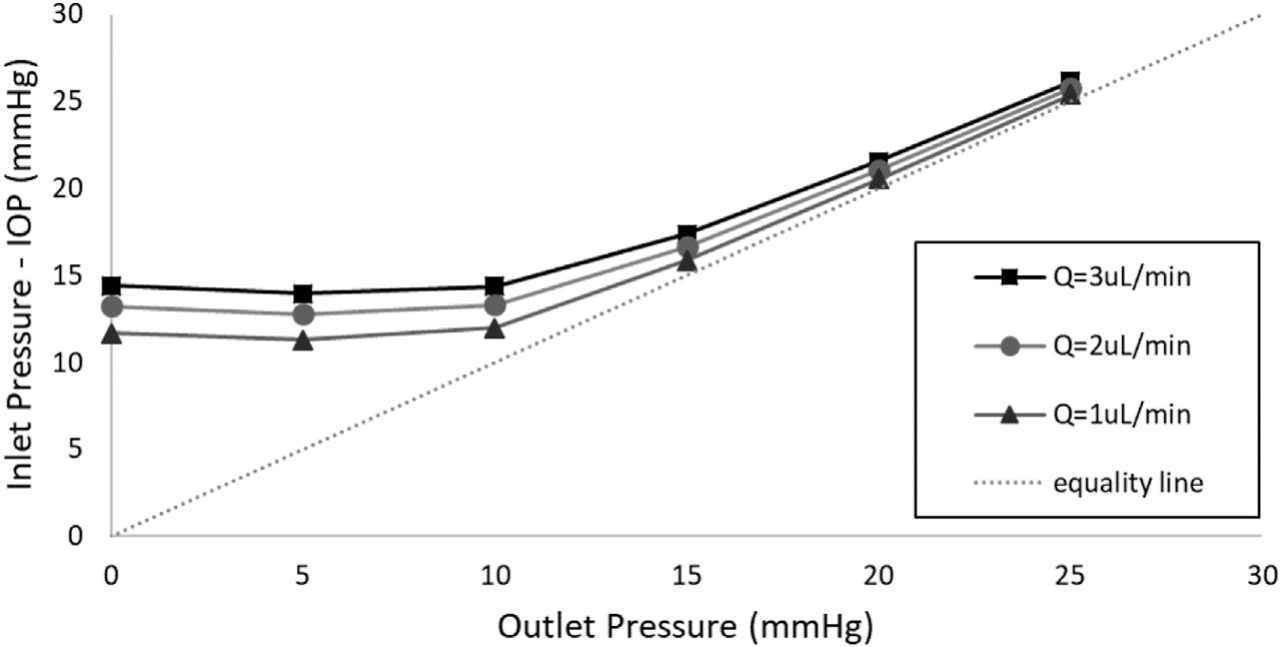
The impact of the flow rate on the valve behavior when a pressure of 7.5 mmHg is applied to the 3 µm thick membrane.

Figure 8 depicts the influence of flow rates set at 1, 2, and 3 μL/min on the valve behavior when an external pressure of 7.5 mmHg is applied to the 3 µm thick membrane. It is evident that increasing the flow rate from 1 to 3 μL/min raises the IOP at each distal pressure, with the magnitude of this difference diminishing as the distal pressure increases. Importantly, this increment, with the highest difference being less than 3 mmHg occurring at 0 distal pressure, is not substantial enough to cause valve dysfunction, leading to either hypertony or hypotony. The figure highlights that, under external pressures of 7.5 mmHg, the valve effectively maintains IOP within the target values across a wide range of flow rate values. This implies that variations in aqueous humor production rates among individuals will not compromise the functionality of the valve, thanks to its robust operating mechanism.

## 4 Discussion

### 4.1 The ideal SAGDD

Here, we have introduced and thoroughly assessed a new SAGDD. This device possesses the unique ability to adapt its resistance to fluid flow in response to changes in the pressure at the valve’s proximal and distal ends. This innovative feature ensures that IOP remains consistently within the physiological range by dynamically altering the cross-sectional area of the fluid pathway within the drainage system, thereby regulating its fluidic resistance in real-time.

In the context of a severe glaucoma-afflicted eye, it can be hypothesized that the typical biological pathways responsible for the drainage of aqueous humor are substantially obstructed and nearly all aqueous humor passes through the SAGDD. As a result, when employing the SAGDD for such patients, the IOP becomes intimately linked to the flow rate traversing through the device and the various fluidic resistances along its path. These impediments encompass both valve and fibrosis resistances that are arranged in series, and their combined effect can be quantitatively expressed using Eq. 4. In this equation, Rf represents fibrosis resistance, Rv signifies valve resistance and Q denotes the flow rate of aqueous humor passing through the implant.

$$IOP=\Delta \text{Pv}+\Delta \text{Pf}=\text{Rv}.Q+\text{Rf}.Q=\left(Rv+Rf\right).Q$$ (4)

During the early post-operative period, prior to the development of fibrosis (Rf is negligible), the distal pressure (Pd) across the drainage valve is close to zero. In this phase, it is imperative for an ideal valve to offer significant resistance (Rv) to maintain IOP above 6 mmHg, thus preventing the occurrence of hypotony. However, as fibrosis progressively surrounds the drainage plate over time, fibrosis resistance increases, resulting in an elevation of the outlet pressure of the valve. Consequently, the resistance of the ideal valve must be adjusted downward to ensure that the overall resistance (comprising Rv and Rf) remains constant. This dynamic adaptation in valve resistance is crucial for long-term ocular health following glaucoma surgery.

As fibrosis resistance progressively increases, it eventually raises the distal pressure to levels above 10 mmHg. Ideally, the valve should exhibit minimal resistance for distal pressures above 10 mmHg, as observed in the valve introduced in this study. However, should fibrosis resistance intensify even further, leading to the distal pressure exceeding the critical threshold of 18 mmHg, it becomes unfeasible to sustain the IOP within the desired target range, even if valve is “fully” open and its resistance is zero.

Our observations reveal that the application of external pressures of 7.5 or 10 mmHg yields an optimally functioning glaucoma valve. This functionality is contingent upon the principle that, when an external pressure is exerted on the valve membrane, it remains in a sealed state as long as the IOP at the inlet remains lower than the applied external pressure. However, as fibrosis gradually develops around the drainage plate, thereby elevating the distal pressure, the hydrostatic force imposed by the aqueous humor on the membrane increases. Consequently, the membrane experiences deformation, causing an expansion in the flow pathway and, in turn, a reduction in resistance to fluid flow. This results in a decrease in inlet pressure (IOP), maintaining it at a relatively constant level.

The experimental results of the ideal SAGDD shown in Figure 7 corroborate and validate the numerical analysis depicted in Figure 6, as they exhibit consistent behavior and trends, albeit with a slight offset between the values. These discrepancies may stem from manufacturing and experimental errors, as well as variations in the exact material properties of the membrane between the prototype and the simulation. Despite these variations, the overall agreement in behavior underscores the reliability of our numerical model and reinforces the confidence in its ability to represent the functionality of the valve accurately. It is important to highlight that Figure 7 was generated based on average values derived from three separate measurements of one valve. Throughout multiple *in vitro* tests, the maximum standard error consistently remained below 0.1 mmHg, a minute deviation that is imperceptible on the diagram.

### 4.2 Design parameters

**FIGURE 9:**
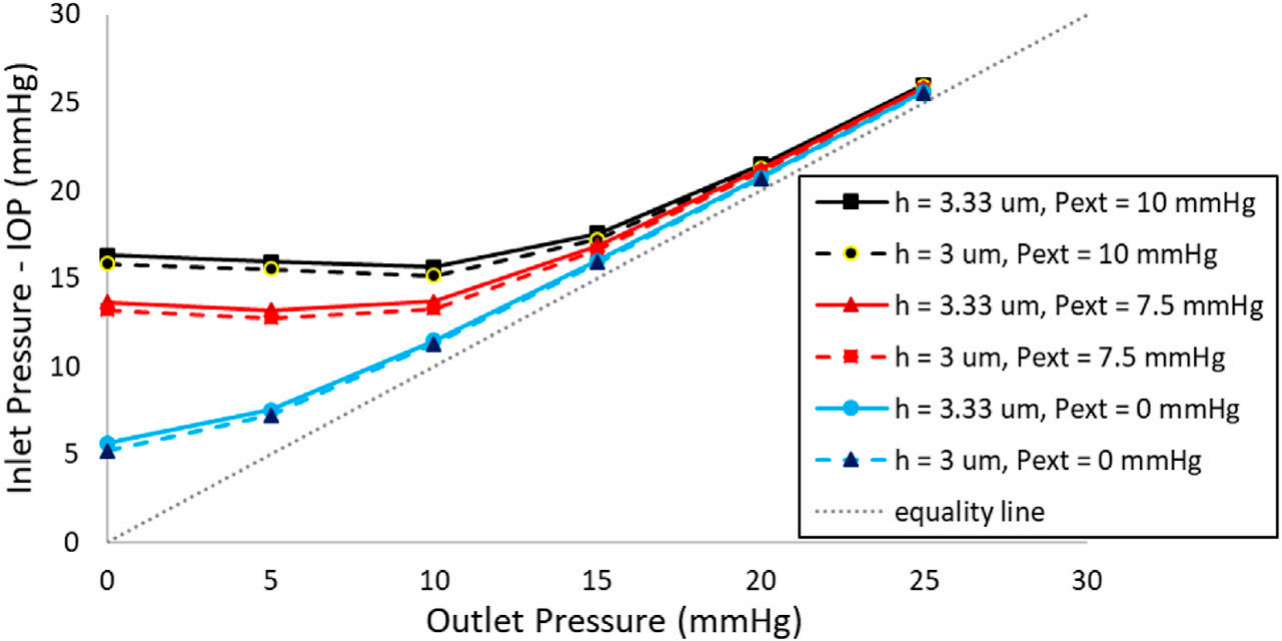
Valve behavior with membrane thickness of 3 µm and 3.33 µm, while all other geometrical conditions remain unchanged.

While the ideal SAGDD introduced in Figure 6 features a membrane with a thickness of 3.33 µm, not readily available from standard metallic membrane manufacturers, an examination of the valve’s behavior reveals that the difference between a 3 μm and 3.33 µm thick membrane is quite small (Figure 9). Specifically, the maximum difference in IOP between the valve with a 3 µm and a 3.33 µm thickness membrane is less than 1 mmHg, notably observed when the distal pressure is set to 0. Consequently, it is anticipated that the real-size valve with a membrane thickness of 3 µm will exhibit a similar behavior.

Among all the design parameters, modifying the membrane thickness to achieve a desired valve response has proven to be the most effective and optimal approach. This is primarily due to its significant impact on the valve’s behavior, allowing us to finely adjust the resistance as needed. In addition to membrane thickness, alternative biocompatible materials, such as titanium, which is softer than stainless steel, can also be considered. Conversely, the situation is different for valve/membrane diameter, as there are constraints on both increasing and decreasing its size. Surgical limitations restrict significant increases, while manufacturing constraints limit significant reductions in size. The smaller the diameter of the valve, the more crucial manufacturing precision becomes, making it a key consideration.

A key advantage of the SAGDD introduced in this study is its ability to function effectively irrespective of the aqueous humor production rate (flow rate). The valve’s capacity to accommodate a range of flow rates ensures reliable performance, making it well-suited for individuals with diverse physiological conditions. This feature enhances the versatility and adaptability of the valve, contributing to its efficacy in addressing the needs of a broad spectrum of patients.

### 4.3 The advantages of the SAGDD

**FIGURE 10:**
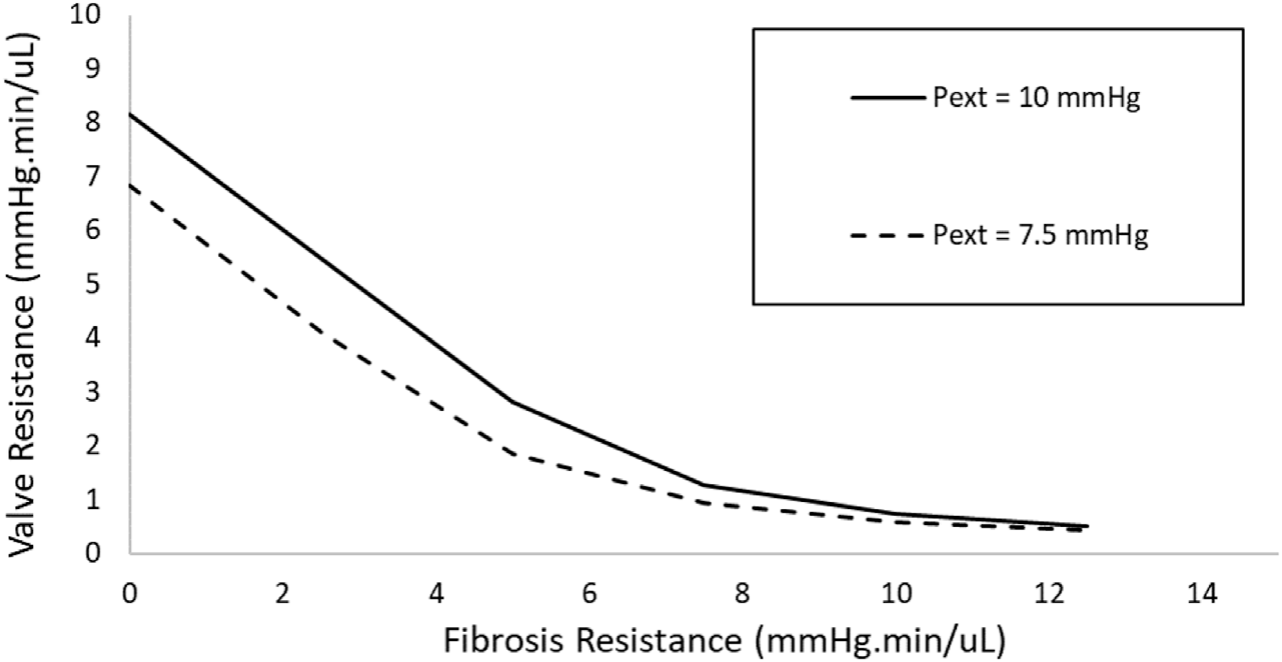
Relationship between valve resistance and fibrosis resistance of SAGDD with 3.33 µm membrane thickness under 7.5 and 10 mmHg external pressures.

While conventional valved implants, such as the Ahmed GDD, work by maintaining a steady fluidic resistance to prevent hypotony, the SAGDD introduced here offers notable advantages. Specifically, the SAGDD is expected to exhibit an extended lifespan compared to other valved implants due to its unique self-response to bleb resistance. Unlike traditional implants with fixed fluidic resistance, our device incorporates a self-adjusting valve mechanism. This mechanism dynamically responds to changes in outlet pressure resulting from bleb formation. As bleb resistance increases, indicating elevated pressure, our valve mechanism reduces its resistance, effectively compensating for the added resistance within the bleb. To illustrate this dynamic response, Figure 10 illustrates the resistance profile of the SAGDD at various levels of fibrosis resistance. As shown, the SAGDD’s resistance decreases with rising fibrosis resistance. In contrast, conventional valved GDDs maintain a static resistance level regardless of fibrosis resistance, potentially leading to premature hypertony due to increased fibrosis resistance. Furthermore, the newly introduced SAGDD presents distinct advantages over the eyeWatch on multiple fronts. Firstly, unlike the eyeWatch, which lacks a self-adjustable valve function, the SAGDD ensures optimal pressure regulation autonomously, eliminating the need for manual adjustments. Secondly, whereas the eyeWatch’s intricate mechanism contributes to high manufacturing costs and results in a relatively expensive end-user price, the design of the SAGDD enables more cost-effective production while maintaining functionality. Overall, the introduction of the cost-effective SAGDD marks a significant advancement in glaucoma management.

### 4.4 Limitations

In this study, we recognize several significant limitations that merit attention. Firstly, the valves were produced at a scale of 3:1 rather than their actual size. This decision was prompted by the considerable difficulties encountered in fabricating and assembling the prototype at its true dimensions. Despite the existence of highly precise micro CNC machines and other micro-manufacturing techniques that could potentially make this feasible, downsizing the device to match real-world proportions introduced complexities in manufacturing processes. These complexities included issues related to precision engineering and material compatibility.

Secondly, while numerical simulations have long been regarded as powerful tools for designing and analysing complex models and validating concepts, it is crucial to acknowledge that the results of this study relied on computational simulations validated with a limited number of valves at a 3:1 scale. It is evident that conducting more extensive *in vitro* and *in vivo* experiments is imperative to assess the functionality of the designs from a real-world perspective. These experiments are necessary to ensure the functional efficacy and safety of the valve in practical applications. Biological factors, such as fibrosis formation, can potentially lead to device failure due to obstructions or malfunctions. Therefore, thorough evaluation through both laboratory and living organism testing is essential to address these concerns and enhance the reliability of the valve’s performance in real-life scenarios.

## 5 Conclusion

In conclusion, our study has established a solid foundation for further exploration of the potential effectiveness of the SAGDD as a viable treatment approach for glaucoma. The SAGDD, with its inherent ability to autonomously regulate pressure and its cost-effective design devoid of expensive instrumentation, holds promise for addressing glaucoma-related challenges. However, it is important to recognize that the outcomes discussed in this study are based on simulations and *in vitro* experiments. To validate and extrapolate these findings, future research should encompass comprehensive *in vivo* investigations involving both animal and human subjects. These forthcoming studies are imperative to bridge the translational gap between laboratory findings and clinical application, thus ensuring the practical feasibility and efficacy of the SAGDD in the management of glaucoma.

## Data Availability

The raw data supporting the conclusion of this article will be made available by the authors, without undue reservation.
